# Analysis of Effect of Intensity of Aerobic Exercise on Cognitive and Motor Functions and Neurotrophic Factor Expression Patterns in an Alzheimer’s Disease Rat Model

**DOI:** 10.3390/jpm13111622

**Published:** 2023-11-20

**Authors:** Do-Youn Lee, Sang-Cheol Im, Na-Yeon Kang, Kyoung Kim

**Affiliations:** Department of Physical Therapy, College of Rehabilitation Science, Daegu University, Gyeongsan 38453, Republic of Korea; triptoyoun@kookmin.ac.kr (D.-Y.L.); marinept83@daum.net (S.-C.I.); skdussla123@naver.com (N.-Y.K.)

**Keywords:** aerobic exercise, Alzheimer’s disease, cognitive function, motor function, neurotrophic factors

## Abstract

The effect of aerobic exercise at different intensities on Alzheimer’s disease (AD) still remains unclear. We investigated the effect of aerobic exercise at different intensities on cognitive and motor functions and neurotrophic factor expression. Thirty-two AD-induced rats were randomly assigned to control (CG), low-intensity (Group I), medium-intensity (Group II), and high-intensity (Group III) exercise groups. Each group, except for the CG, performed aerobic exercise for 20 min a day five times a week. After performing aerobic exercise for 4 weeks, their cognitive and motor functions and neurotrophic factor expression patterns were analyzed and compared between the groups. All variables of cognitive and motor functions and neurotrophic factor expression were significantly improved in Groups I, II, and III compared to those in the CG (*p* < 0.05). Among the neurotrophic factors, brain-derived neurotrophic factor (BDNF) expression was significantly improved in Group III compared to that in Groups I and II (*p* < 0.05). In the intra-group comparison of cognitive and motor functions, no significant difference was observed in CG, but the aerobic exercise groups showed improvements. Only Group III showed a significant improvement in the time it took to find eight food items accurately (*p* < 0.05). Aerobic exercise improved the cognitive and motor functions and neurotrophic factor expression patterns in the AD-induced rat model, with high-intensity aerobic exercise having greater effects on cognitive function and BDNF expression.

## 1. Introduction

Alzheimer’s disease (AD) is among the most common neurodegenerative disorders, and it is characterized by a progressive decline in cognition, behavior, and the ability to perform the activities of daily living [1]. As life expectancy increases, AD incidence also continues to increase [2], and this has a very serious impact on the physical and mental health of patients and caregivers, as well as considerable socioeconomic impacts [3]. The progressive onset and slow worsening of AD can be explained by key biomarkers [4]. The accumulation of β-amyloid protein in the brain, the degeneration or damage of nerves, increased tau protein in the cerebrospinal fluid, decreased uptake in the temporoparietal cortex, and atrophy of the temporal lobe and medial parietal cortex are all observed in AD patients [5].

Neurotrophic factors are closely involved in neuronal generation, differentiation, and survival [6]. In neurodegenerative diseases such as AD, the expression of neurotrophic factors is reduced due to β-amyloid deposition in the brain, resulting in cognitive decline [7]. This cognitive impairment causes motor function problems, even when there is no problem in the musculoskeletal system [8]. Studies on the factors that regulate the developmental processes of nerve cells, including neurotrophic factors, provide important information for understanding and treating cranial nerve diseases [9]. A neurotrophic factor is a protein that regulates the regeneration and growth of brain nerve cells, and they are important for the development and maintenance of the central nervous system [10,11]. These neurotrophic factors include insulin-like growth factor-1 (IGF-1), nerve growth factor (NGF), and brain-derived neurotrophic factor (BDNF).

Several recent studies have suggested potential advancements in the treatment methods used for degenerative brain diseases [12,13,14]. However, there are limitations to AD prevention and treatment methods because the available treatments mainly focus on alleviating symptoms [15]. Physical activity reduces the risk of AD and improves the physical functioning of AD patients, allowing them to perform the activities of daily living [16,17]. Additionally, physical activity modifies the pathological process of AD and maintains cognitive function [18]. Among the effective physical activities, aerobic exercise is the most promising and cost-effective strategy for delaying or preventing AD [19]. The beneficial effects of aerobic exercise on AD patients and AD animal models have been reported in recent studies [20,21,22,23]. However, some studies found that aerobic exercise had no effect [24,25]. These studies found a lack of association between physical activity and cognitive decline and suggested that the benefits of exercise for the brain may be related to non-amyloid effects.

As such, the effect of aerobic exercise on AD remains unclear and controversial. These conflicting results may be due to differences in the research methodologies used in the studies. Thus far, the effects of different intensities of aerobic exercise on AD patients has not been studied, and there are limitations when assessing the effects of aerobic exercise on the pathophysiology of AD in humans. Therefore, this study aimed to investigate the effect of aerobic exercise at different intensities on cognitive and motor functions and neurotrophic factor expression patterns in an AD-induced rat model. We hypothesized that different intensities of aerobic exercise would affect cognitive and motor functions and neurotrophic factor expression patterns in the AD-induced rat model in different ways.

## 2. Methods

### 2.1. Experimental Animals

**Figure 1:**
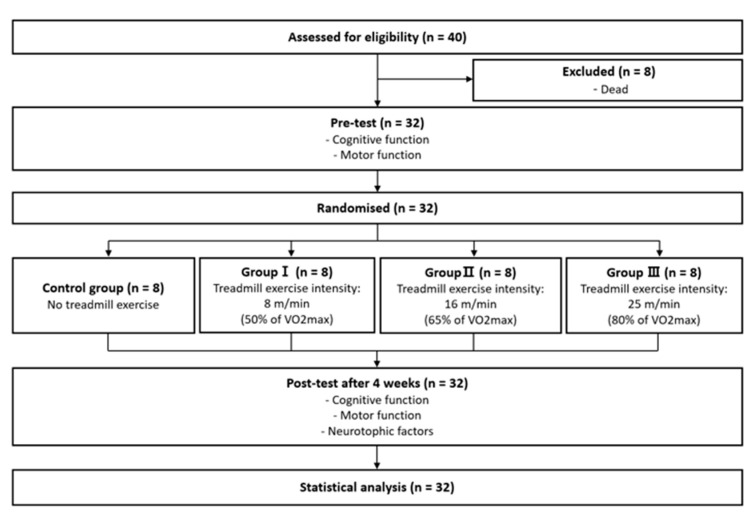
CONSORT diagram presenting the study flow.

Our animal experiments were approved by the Animal Care Committee of Daegu University (DUIACC-2017-9-0216-007). Forty male Sprague Dawley rats aged 10 weeks were used in this study. Thirty-two of the animals were used in the experiments, while eight of the animals were killed immediately after AD induction. The rats were numbered and randomized using an Excel random draw method. The rats were randomly assigned to control (CG, n = 8), low-intensity (Group I, n = 8), medium-intensity (Group II, n = 8), and high-intensity (Group III, n = 8) exercise groups (Figure 1). All the rats had free access to standard food (Cargill Agri Purina, Seongnam, Republic of Korea) and water in a controlled environment (12 h light/dark cycle, 23 ± 2 °C with 50% relative humidity) [26].

### 2.2. Induction of Alzheimer’s Disease

Zoletil (Virbac Laboratories, Carros, France) and Rompun (Bayer, Seoul, Republic of Korea) were mixed at a ratio of 1:1, and 2 mL per 1 kg of body weight was intraperitoneally injected into the rat to induce general anesthesia. STZ (30 μg μL-1) was dissolved in sodium citrate (pH 4.2, 1% *w*/*v*) and prepared before injection. To prepare the sodium citrate buffer, 2.1 g of citric acid (FW: 210.14) and 2.94 g of sodium citrate (FW: 294.10) were diluted in 100 mL of distilled water to form liquid A and liquid B, respectively. Liquid A and liquid B were mixed 1:1 to form the sodium citrate buffer pre-use [27]. The head of the rat was then fixed to a stereotaxic frame and the skull was shaved. After disinfecting the incision, streptozotocin (STZ) was administered via intracerebroventricular (ICV) injection to induce AD. The ICV injection of STZ into a rat results in the accumulation of β-amyloid in the brain, and this is consistent with the biochemical changes that occur in the brains of AD patients [28]. A hole was drilled using a small drill with a diameter of 0.5 mm at a point 1.5 mm to the right and 0.8 mm to the rear of the bregma on the surface of the rat’s skull. A 26-G syringe (Hamilton, Reno, NV, USA) was then inserted to a depth of 3.6 mm to inject 20-μL of STZ [29]. The other side of the skull was injected in the same manner. All operations were performed under aseptic conditions. After suturing the incision with silk suture, it was disinfected, and the rat was returned to the laboratory.

### 2.3. Treadmill Exercise Protocol 

After confirming AD induction, all of the rats went through an acclimatization period of 7 days before beginning the exercise. A rodent treadmill (Jeungdo, Seoul, Republic of Korea) was used for the aerobic exercise. Treadmill exercise was undertaken by all groups except the CG at an inclination of 0° for 20 min a day, five times a week, for 4 weeks. The treadmill exercise was performed during the dark part of the time cycle, when the rats’ activity increases, so that effective exercise could be performed according to the rats’ biological rhythm. To determine the appropriate exercise intensity, the results of previous studies that measured maximum oxygen consumption (VO2max) according to treadmill speed in rats were used as references [30,31]. Thus, the exercise intensity in Group I was 8 m/min, corresponding to approximately 50% of their VO2max; the exercise intensity in Group II was 16 m/min, which was approximately 65% of their VO2max; and the exercise intensity in Group III was 25 m/min, which was approximately 80% of their VO2max.

### 2.4. Cognitive Function Test

#### Eight-Arm Radial Maze Test

The 8-arm radial maze test measures working ability and spatial memory, which correspond to the rats’ cognitive function. The tool had a radial shape and comprised eight passages with a size of 12 × 60 cm leading from the central starting point. Each maze is 30 cm high to prevent the rats from escaping. Food, which was provided as a reward, was placed at the end of a passage (arm), and learning was conducted after the rats were fasted for 24 h before entering the maze. After learning, the rats were fasted again for 24 h and then tested. In the test, they were allowed to move freely through the maze and eat any food they found at the end of a passage. The time the experimental animals took to accurately find food in all eight passages within 5 min was measured. Repeatedly entering the same passage was evaluated as an error, and the number of times this happened was measured; the number of times the rat accurately searched for food before the first error occurred was also measured.

### 2.5. Motor Function Test

#### 2.5.1. Beam-Walking Test

The beam-walking test evaluates sensory and motor functions by showing how well an experimental animal can walk on a narrow bar. The movement of the rats as they crossed a narrow wooden bar with a length of 80 cm and a width of 5 cm was evaluated. A total of three tests were performed, and the average value was used. Scores ranged from 0 to 6 points. Referring to previous studies, “the state of being unable to balance on a bar and maintaining it for 10 s” was scored as 0, and “the state of crossing a narrow bar without slipping of the feet” was scored as 6 [32].

#### 2.5.2. Ladder Rung Walking Test

The ladder rung walking test measures deficits in and recovery of kinesthetic function. A ladder with a spacing of 1.5 cm was placed between horizontal bars with a length of 10 cm and a width of 10 cm. Rat breeding farms were present at the start and end of the ladder, and they were installed at a height of approximately 30 cm from the ground. The failure rate (i.e., the rate at which the leg of the rat fell under the ladder) was measured by using a video camera to record the rat crossing the ladder. A score of 0 was given for “the state of losing one’s body posture and balance by dropping one’s feet between the ladders”, and a score of 6 was given for “the state of stepping on the middle part of the ladder well and fully bearing weight” [33]. An error was defined as a score of 0, 1, or 2, and the error score was expressed as a value converted into a percentage by dividing the total number of foot errors by the number of counted steps.

### 2.6. Histological Examination

#### 2.6.1. Brain Extraction and Tissue Fixation

Brain tissue was extracted for Western blot analysis after a 4-week treadmill exercise program. To collect the brain tissues from each group of experimental animals, 1 mL of tribromoethanol per 1 kg of body weight was injected intraperitoneally to induce general anesthesia. Thereafter, myocardial perfusion was performed using a 0.9% NaCl solution to remove blood from the body. The brain was quickly removed, and the motor cortex was isolated on ice. The samples were flash frozen on dry ice and stored at −80 °C.

#### 2.6.2. Western Blot Analysis

For the immunohistochemical analysis, the immune responses to the neurotrophic factors IGF-1, NGF, and BDNF were observed using Image-proplus ver 4.0 (Media Cybernetics, Rockville, MD, USA) by connecting a computer to a CCD camera (Toshiba, Kawasaki, Japan) mounted on an Olympus BX50 optical microscope (Olympus, Tokyo, Japan). Altogether, 30 μg of total protein was extracted from the brain tissue and electrophoresed in a 10% SDS-polyacrylamide gel at 80 volts, and then separated with a nitrocellulose membrane for 60 min. After separation, the membrane was blocked with 3% skim milk solution for 1 h on a rocker platform. The primary antibodies—anti-BDNF (Santa Cruz Biotechnology, Santa Cruz, CA, USA) and antiNGF (Santa Cruz Biotechnology)—were then diluted 1:1000 in a 3% skim milk solution and shaken for 12 h. Next, after washing with a TBS-T solution five times for 10 min each, the secondary antibody (Zymed, San Francisco, CA, USA) was diluted 1:5000 with a blocking solution and shaken for 1 h. After it was rinsed with the TBS-T solution five times for 10 min each, in the last step, the membrane was placed into a WBLR solution (Santacruz Biotechnology) and colored for 1 min. Finally, the obtained membrane was scanned using the Molecular Imager ChemiDoc XRS System (Bio-Rad, Hercules, CA, USA) for image analysis. The amount of protein was then calculated using the Quantity One 1-D Analysis Software (Bio-Rad, Hercules, CA, USA).

### 2.7. Statistical Analysis

All data were analyzed using the IBM SPSS for Windows version 22.0 software (IBM Corp., Armonk, NY, USA). All values are expressed as mean ± standard deviation. A normal distribution analysis was performed using the Shapiro–Wilk test, and all the data showed a normal distribution. One-way analysis of variance was performed to analyze the differences between the groups, and the least significant difference test was used as a post-hoc test. A paired *t*-test was conducted to compare the effects before and after exercise within each group. The statistical significance level (α) was set at 0.05.

## 3. Results

### 3.1. Comparison of Cognitive Function Results between and within Groups 

**Figure 2:**
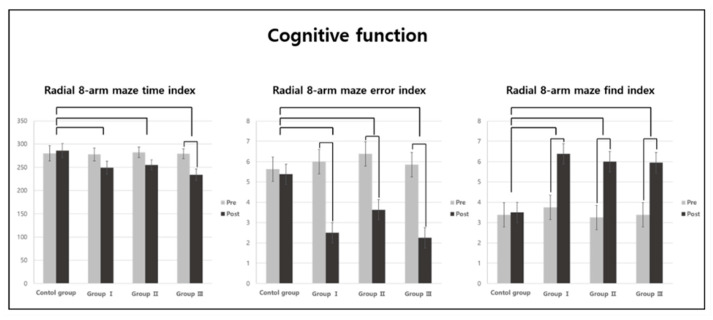
Post-hoc analysis of cognitive function variables.

There were significant differences in all variables in the comparison of the 8-arm radial maze test results for each group (*p* < 0.05; Table 1). A post-hoc analysis showed that the time taken to find eight food items as well as the error index values (i.e., the number of times a rat found the same food item) were significantly lower in Groups I, II, and III than in the CG (Figure 2). The number of times food was found accurately before the first error occurred was significantly higher in Groups I, II, and III than in the CG (Figure 2).

In the comparison within the groups, the time taken to find eight food items was significantly shorter only in Group III, and the number of errors was significantly lower in Groups I, II, and III (*p* < 0.05; Table 1). The frequency with which food was found accurately before the first error occurred was significantly higher in Groups I, II, and III (*p* < 0.05; Table 1).

### 3.2. Comparison of Motor Function Results between and within Groups

**Figure 3:**
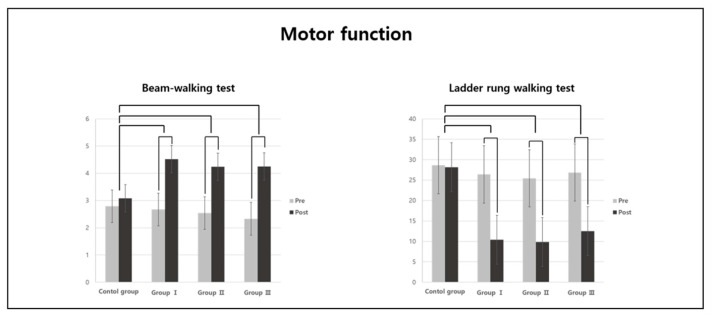
Post-hoc analysis of motor function variables.

There were significant differences in the beam-walking and ladder rung walking test results among the groups (*p* < 0.05; Table 2). As a result of the post-hoc analysis, the beam-walking test scores were significantly higher in Groups I, II, and III than in the CG (Figure 3). The ladder rung walking test scores were also significantly higher in Groups I, II, and III than in the CG (Figure 3).

In the comparison within the groups, the beam-walking test scores were significantly higher in Groups I, II, and III than in the CG (*p* < 0.05; Table 2). The ladder rung walking test scores were also significantly higher in Groups I, II, and III than in the CG (*p* < 0.05; Table 2).

### 3.3. Comparison of Neurotrophic Factor Expression Results between Groups

**Figure 4:**
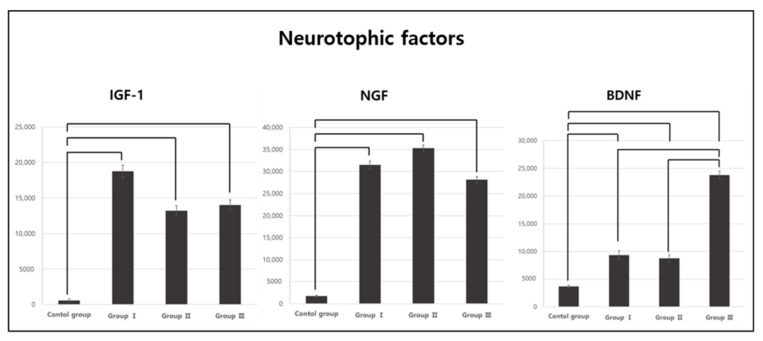
Post-hoc analysis of the neurotrophic factor variables.

There were significant differences in the expression of the neurotrophic factors IGF-1, NGF, and BDNF among the groups (*p* < 0.05; Table 3). The post-hoc analysis showed that the expression of IGF-1 and NGF was significantly higher in Groups I, II, and III than in the CG (Figure 4). The BDNF expression was also significantly higher in Groups I, II, and III than in the CG, and it was significantly higher in Group III than in Groups I and II (Figure 4).

## 4. Discussion

We investigated the effect of aerobic exercise at different intensities on cognitive and motor functions and neurotrophic factor expression in an AD-induced rat model. AD was induced by administering ICV STZ in rats, and the rats undertook aerobic exercise at different intensities for 4 weeks. Afterward, their cognitive function, exercise behavior, and histological test results were used to compare and analyze the effects of the different intensities of aerobic exercise.

In the present study, an 8-arm radial maze test was conducted to evaluate the spatial memory and work ability of the AD rats to investigate the effect of aerobic exercise on their cognitive function. In the comparison of the 8-arm radial maze test results between the different groups, all three cognitive function variables improved significantly in Groups I, II, and III compared to those observed in the CG (the rats in the CG did not undertake aerobic exercise). However, there was no difference in the intensity of aerobic exercise among the groups. Many recent studies have reported the positive effects of aerobic exercise on cognitive function in AD-induced rat models [34,35,36] and AD patients [23,37,38]. Farzi et al. [34] reported that cognitive ability and memory improved after 8 weeks of aerobic exercise in an AD-induced rat model. Mu et al. [35] reported that 12 weeks of aerobic exercise improved the spatial working memory of AD rats. Additionally, Dare et al. [36] reported that 4 weeks of aerobic exercise led to memory enhancement, especially in long-term object recognition memory, in an AD-induced rat model. Sobol et al. [23] also showed that aerobic exercise improved the VO2peak of patients with mild AD, and that the changes in VO2peak are positively correlated with improved cognitive function. Yu et al. [37] demonstrated that 6 months of aerobic exercise could reduce the overall cognitive decline among elderly people with AD dementia. Yang et al. [38] demonstrated that moderate-intensity aerobic exercise improves the cognitive function of patients with mild AD. As such, previous studies that have reported the positive effects of aerobic exercise on cognitive function in AD-induced rat models and AD patients have shown similar results to those obtained in the present study. Physical activity increases the brain’s neuroplasticity, and this is effective in improving cognitive ability and memory [39]. Aerobic exercise in particular is associated with improved cognitive function because it reduces Aβ protein deposition and improves the insulin signaling pathway [40]. Therefore, we believe that our aerobic exercise groups developed enhanced brain neuroplasticity and underwent hippocampal nerve regeneration through regular physical activity. Additionally, in the intra-group comparison of the time taken to find the eight food items, only Group III showed a significant improvement. Thus, high-intensity aerobic exercise has a greater positive effect on cognitive function than the other exercise intensities.

The beam-walking and ladder rung walking tests were conducted to measure the motor function in the AD-induced rat model. The beam-walking test is a method for evaluating the sensory and motor functions as well as balance [32]. The ladder rung walking test measures aging, motor system deficits, and functional recovery [41]. In the present study, motor function was significantly improved in Groups I, II, and III compared to the CG according to both tests. However, there was no difference in the intensity of the aerobic exercise. In the comparison within the groups, Groups I, II, and III showed a significant improvement after the aerobic exercise compared with before. In previous studies, motor function, which can be reduced due to various diseases that cause brain damage, such as AD and stroke, can be improved through aerobic exercise, such as walking and swimming [42,43]. Additionally, it has been shown that a decline in motor function after a brain injury has a significant effect on decreases in cognitive function and muscle strength, and that aerobic exercise is effective in restoring sensory and motor functions following brain damage [44]. In the present study, regular and continuous treadmill exercise was undertaken by the test subjects as an aerobic exercise, and it improved the coordination ability of the motor-sensory system in the AD-induced rat model. Physical exercise helps in the recovery of movement and motor function, and it is also closely related to the improvement of cognitive function [45]. In this study, both cognitive and motor functions were improved after aerobic exercise, and the findings of previous studies were thus supported.

Neurotrophic factors are a family of growth factors which are required for the survival and dendrite formation of specific neuronal populations during brain development [46,47]. They can protect the aging hippocampus, an area involved in learning and memory, by preventing neuronal death and axonal degeneration [48]. Among the neurotrophic factors, IGF-1 encodes a regulatory protein that is involved in a series of intracellular biological actions ranging from DNA synthesis to cell circulation and death [49]. It also plays a neuroprotective role and improves motor and cognitive functions [50]. Subnormal levels of IGF-1 are found in the brain and blood in patients with neurodegenerative diseases, especially AD [51]. NGF is a protein necessary to maintain the growth and function of sympathetic nerve neurons. Promoting NGF secretion through exercise activates the synapses and thereby improves memory and judgment and increases the recovery rate of damaged brains, such as those of AD and stroke patients [52]. BDNF is involved in neurogenesis and memory functions, including neural connectivity, synaptic development, and plasticity. BDNF is reported to be an important regulator of synaptic transmission and long-term potentiation in the hippocampus and other brain regions [53]. Additionally, increasing evidence has shown that the BDNF protein is reduced in AD patients and AD animal models [54,55].

In this study, IGF-1 expression was significantly higher in the CG than in Groups I, II, and III, but no difference was found among the rats when stratified according to aerobic exercise intensity. Changes in IGF-1 levels were found to correlate positively with changes in hippocampal volume and late verbal recall performance following 3 months of aerobic exercise in healthy elderly people [56]. Another study reported that the concentration of IGF-1 increased in the prediabetic elderly (a risk factor for AD) following 6 months of aerobic exercise [57]. These results are consistent with the results of the present study, which showed that the IGF-1 level significantly increased in the aerobic exercise group compared to the CG.

NGF expression was significantly increased in Groups I, II, and III compared to the CG, but there was no difference among the rats when stratified according to aerobic exercise intensity. Um et al. [58] reported an increase in NGF expression after 12 weeks of aerobic exercise in an AD-induced rat model. Radak et al. [59] showed an increase in NGF expression after 12 weeks of aerobic exercise in a stroke rat model. Additionally, Ding et al. [60] showed that, as a result of undertaking aerobic exercise, brain damage symptoms were improved due to increased NGF expression in a rat model of middle cerebral artery occlusion. The results of previous studies and those of this study were therefore similar.

In the present study, BDNF expression was significantly increased in Groups I, II, and III compared to the CG. Previous studies have suggested that physical activity can play a role in improving cognitive function by inducing the upregulation of BDNF expression in the hippocampus and cerebral cortex [58,61]. Another study demonstrated that motor function training and aerobic exercise in rats increased BDNF expression in the cerebellum [62]. Moreover, BDNF expression was significantly higher in Group III than in Groups I and II. In a previous study on BDNF expression among groups stratified according to the intensity of aerobic exercise, BDNF expression was increased in the high-intensity exercise group compared to the healthy adult men engaged in low-intensity cycling exercises [63]. Additionally, the group engaged in cycling exercise at a 70% VO2max intensity for 30 min showed a higher BDNF concentration than the group that exercised at a 55% VO2max intensity [64], indicating that high-intensity aerobic exercise more effectively increases BDNF expression, thereby supporting the results of this study.

This study has several limitations. First, the effect of exercise frequency was not considered. In future studies, investigations into the complex action of aerobic exercise intensity and frequency will be needed. Second, the effect of aerobic exercise according to AD onset period was not investigated. In the future, it will be necessary to classify the subjects according to AD onset period and study the relationship between AD onset period and aerobic exercise. Third, we did not evaluate whether the effect of aerobic exercise was sustained in the long term.

## 5. Conclusions

Aerobic exercise of all intensities improved cognitive function, motor function, and neurotrophic factor expression in the AD rat model. No differences were found in motor function, IGF-1 expression, or NGF expression in the groups that undertook aerobic exercise of different intensities, but high-intensity aerobic exercise had a significant effect on cognitive function and BDNF expression. The results of this study suggest the need for further research on the effects of differences in aerobic exercise intensity.

## Figures and Tables

**Table 1:** Comparison of cognitive function results between and within groups.

Variable	Control Group		Group I		Group II		Group III		F (*p*)
	Pre	Post	Pre	Post	Pre	Post	Pre	Post	
Radial 8-arm maze time index (second)	280.25 (16.49)	286.12 (15.18)	277.63 (13.96)	249.13 (14.04)	282.13 (11.25)	255.32 (10.45)	279.38 (10.61)	234.13 ^‡^ (12.18)	33.631 (0.025 *)
Radial 8-arm maze error index (number)	5.63 (1.69)	5.38 (1.52)	6.00 (1.69)	2.50 ^‡^ (1.31)	6.38 (1.41)	3.63 ^‡^ (1.18)	5.85 (1.04)	2.25 ^‡^ (1.26)	8.893 (0.000 *)
Radial 8-arm maze find index (number)	3.48 (0.52)	3.50 (0.73)	3.75 (0.46)	6.38 ^‡^ (0.52)	3.25 (0.84)	6.00 ^‡^ (0.76)	3.38 (0.63)	5.95 ^‡^ (0.46)	9.241 (0.000 *)

* *p* < 0.05 is significant in ANOVA; ^‡^ *p* < 0.05 is significant in paired *t*-test.

**Table 2:** Comparison of motor function results between and within groups.

Variable	Control Group		Group I		Group II		Group III		F (*p*)
	Pre	Post	Pre	Post	Pre	Post	Pre	Post	
Beam-walking test (score)	2.79 (0.42)	3.08 (0.61)	2.67 (0.87)	4.52 ^‡^ (0.24)	2.54 (0.78)	4.24 ^‡^ (0.38)	2.33 (0.36)	4.25 ^‡^ (0.39)	6.367 (0.002 *)
Ladder rung walking test (score)	28.65 (4.48)	28.13 (5.34)	26.40 (5.87)	10.42 ^‡^ (4.26)	25.44 (4.16)	9.86 ^‡^ (3.92)	26.87 (5.65)	12.50 ^‡^ (3.15)	9.890 (0.000 *)

* *p* < 0.05 is significant in ANOVA; ^‡^ *p* < 0.05 is significant in paired *t*-test.

**Table 3:** Comparison of neurotrophic factor expression results between groups.

Variable	Control Group	Group I	Group II	Group III	F (*p*)
IGF-1 (pixels)	566.00 (220.26)	18,782.75 (835.37)	13,213.25 (691.47)	14,016.63 (719.59)	1261.93 (0.000 *)
NGF (pixels)	1728.25 (198.86)	31,503.50 (1253.11)	35,315.63 (1456.08)	28,126.88 (955.53)	1015.31 (0.000 *)
BDNF (pixels)	3672.25 (400.90)	9311.50 (939.24)	8733.50 (736.01)	23,787.51 (916.93)	987.89 (0.000 *)

* *p* < 0.05.

## Data Availability

The data presented in this study are available on request from the corresponding author. The data are not publicly available due to ethical restrictions.
